# Disulphide bond reduction of a therapeutic monoclonal antibody during cell culture manufacturing operations

**DOI:** 10.1186/1753-6561-5-S8-P110

**Published:** 2011-11-22

**Authors:** Brian Mullan, Bryan Dravis, Amareth Lim, Ambrose Clarke, Susan Janes, Pete Lambooy, Don Olson, Tomas O’Riordan, Bruce Ricart, Alexander G Tulloch

**Affiliations:** 1 Manufacturing Science and Technology, Eli Lilly & Co, Kinsale, Cork, Ireland; 2 Bioprocess R&D, Eli Lilly & Co, Indianapolis, Indiana, USA; 3 Bioproduct Analytical Chemistry, Eli Lilly & Co, Indianapolis, Indiana, USA; 4 Analytical Technical Operations, Eli Lilly & Co, Kinsale, Cork, Ireland

## Background

Disulphide bonding is critical to maintaining immunoglobulin (IgG) tertiary and quaternary structure for therapeutic monoclonal antibodies (MAb). Both inter- and intra-chain disulphide bonds are formed intracellularly in the expression host prior to secretion and purification during MAb production processes. Disulphide bond shuffling has previously been reported for IgG_2_[1,2] and disulphide-mediated arm-exchange for IgG_4_[3,4], reflecting innate behaviour of these IgG classes. However, atypical and significant reduction of disulphide bonds has been recently observed in IgG_1_[5,6] that present significant issues for manufacturing of therapeutic MAbs.

During manufacturing of preliminary lots of a recently transferred MAb manufacturing process (IgG_1_), gross disulphide bond reduction following affinity capture chromatography of clarified production bioreactor material was observed. Investigations leading to the identification of the nature of this reduction process, and process steps to mitigate against its future occurrence, are described here. The MAb was co-developed with MacroGenics, Rockville, MD.

## Methods

Production Bioreactor material for downstream processing was supplied from a 16 day, fed-batch, GS-CHO culture [7]. The Production Bioreactor was a single-use (Wave System) with a 100L (full scale) or 10L (lab model) working volume. Clarified harvest intermediate (CHI) hold studies were performed in either 560L LevMix units (full scale) or 5L Braun benchtop bioreactors (lab model). Purification to produce Affinity Capture chromatography eluted mainstream was performed using a 20cm x 20cm MAbselect Protein A resin (GE Healthcare) column, and an AKTA Process skid (GE Healthcare).

LC-MS analysis was performed on a Polymer Laboratories PLRP-S HPLC column and analyzed using an Agilent 1100 HPLC system coupled to an Applied Biosystems QSTAR XL mass spectrometer, following sample preparation. CE-SDS analysis was performed using a Beckman Coulter PA800 capillary electrophoresis instrument fitted with bare-fused silica capillary and UV detection at 220 nm, following sample preparation. Microchip CE-SDS analysis was performed using a Lab-on-chip microanalyser (Agilent). Free thiols were quantified using Ellman’s reagent. Metabolic analysis was conducted by Metabolon (Durham, NC).

## Results

### Identification of disulphide reduction of IgG during Primary Recovery

The IgG manufacturing process as transferred from the co-developing partner included a cell culture settling step following the Production Bioreactor and prior to Primary Recovery.

Disulphide bond reduction was first detected during initial development runs by routine Non-reduced (NR) CE-SDS in-process analysis after Affinity capture chromatography (data not shown). NR-CE-SDS analysis identified elevated levels of free light chain and half antibody molecules, when compared to Reference Standard.

Additional analysis, employing microchip-based NR-CE-SDS methods indicated that the antibody reduction occurred during the primary recovery cell settling step (results not shown). This was confirmed by LC-MC analysis (results not shown). Assessment of disulphide bonding pattern and intactness by LC-MS peptide mapping identified both inter- and intra-chain disulphide scrambling (results not shown).

### Delineation of events leading to IgG reduction

**Figure 1:**
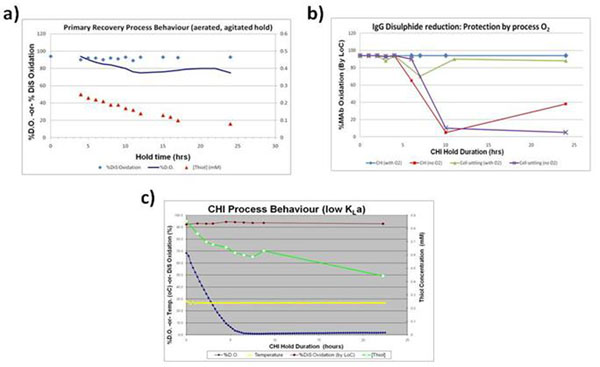
IgG disulphide bond reduction under various conditions for cell-settled and immediately clarified harvest material. CHI, Clarified harvest intermediate; DiS, Disulphide; LoC, Lab-on-a-Chip (Agilent).

Initial investigations to understand process behaviour during primary recovery identified that reducing species, including free thiols (which increase over the course of the Production Bioreactor, up to 1mM), were present at the end of the Production Bioreactor (Figure 1a). Dissolved oxygen was also shown to deplete during the cell settling phase following harvest (data not shown). From this, an initial working hypothesis was formed that reducing species, including free thiols, became reactive at low dissolved oxygen concentrations and led to IgG_1_ disulphide bond reduction.

A revised process control strategy was implemented (see below) to prevent oxygen depletion and maintain dissolved oxygen levels above a minimum level. This involved including an aerated and agitated hold for Clarified Harvest Intermediate (CHI) in the process.

Further studies identified that O_2_ is critical to maintaining a stable environment for oxidised (i.e., normally disulphide bonded) IgG_1_ in CHI. When O_2_ was present, IgG_1_ remained intact under all conditions evaluated. Only when O_2_ was deliberately absent, or stripped away, would the harvest material or CHI demonstrate potential for reduction (Figure 1b).

### Metabolic behaviour of reducing intermediates

The working hypothesis was that by maintaining sufficient levels of dissolved oxygen in the CFM, the thiol species could be reacted out (oxidised) and a stable environment for oxidised IgG_1_ created (Figure 1a). However, the relationship between IgG reduction and thiol redox state is not first order (Figure 1c), and the rate of thiol oxidation was found to be dependent on the source of Production Bioreactor material (i.e., varied with different harvest lots). This indicated the involvement of an additional component, potentially catalytic, which has not yet been identified in our studies. Thioredoxins have been identified as such a catalytic component by others [5,6], and these need to be recycled after one redox cycle via Thioredoxin Reductase / NADP(H).

Metabolic analysis of cell and media material from Production Bioreactors indicated high levels of oxidised homocysteine and cysteine (both reactive redox molecules), which correlated with decreasing levels of folate (B6) and cobalamine (B12), both of which are involved in recycling homocysteine. Overall, this analysis identified numerous options for media optimisation to mitigate against IgG reduction. However, given the success of process controls (described below), and the late stage of process development (pre-validation) these media optimisation options were not pursued.

### Process controls to mitigate disulphide reduction of IgG

A process control strategy was implemented including:

• Establishing a minimal dissolved oxygen level in the Production Bioreactor prior to harvesting

• Immediately clarifying the Production Bioreactor material (i.e., eliminating the cell settling step)

• Holding the CHI in a hold vessel (LevMix container, agitated hold) that had been partially pre-filled with process air.

## Conclusions

• Gross disulphide bond reduction was observed during late stage development of an IgG_1_ monoclonal antibody being commercialised for a therapeutic indication;

• Disulphide bond reduction had a second, or higher, order link to low dissolved oxygen levels in process intermediates, and the involvement of a catalytic factor was also indicated;

• Implementation of an appropriate control strategy (and associated process analytics) informed by process development has ensured no recurrence of this issue (for n=15 full scale lots).
